# Non-targeted discovery of high-value bio-products in *Nicotiana glauca* L: a potential renewable plant feedstock

**DOI:** 10.1186/s40643-023-00726-4

**Published:** 2024-01-18

**Authors:** Natalia Carreno-Quintero, Takayuki Tohge, Rebecca Van Acker, Lauren S. McKee, Qi Zhou, Antje Bolze, Xiaohui Xing, Merve Özparpucu, Markus Rüggeberg, Thomas Piofczyk, Yaw Koram, Vincent Bulone, Wout Boerjan, Alisdair R. Fernie, Paul D. Fraser

**Affiliations:** 1 Biochemistry Department, Royal Holloway University of London, Egham Hill, Egham, Surrey TW20 0EX UK; 2 Max-Planck-Institute of Molecular Plant Physiology, Am Mühlenberg 1, 14476 Potsdam-Golm, Germany; 3 Department of Plant Biotechnology and Bioinformatics, Ghent University, Technologiepark 927, 9052 Ghent, Belgium; 4 Center for Plant Systems Biology, VIB, Technologiepark 927, 9052 Ghent, Belgium; 5 Division of Glycoscience, School of Biotechnology, Royal Institute of Technology (KTH), AlbaNova University Centre, 106 91 Stockholm, Sweden; 6 Division of Glycoscience, School of Biotechnology, Wallenberg Wood Science Centre, KTH, Stockholm, Sweden; 7 Institute for Building Materials, Swiss Federal Institute of Technology Zürich (ETH Zürich), Zurich, Switzerland; 8 Applied Wood Materials, Swiss Federal Laboratories of Materials Science and Technology (EMPA), Dübendorf, Switzerland; 9 Pilot Pflanzenöltechnologie Magdeburg e. V. (PPM e. V.), Berliner Chaussee 66, 39114 Magdeburg, Germany; 10 Neutral Supply Chain Limited, 337 Bath Road, Slough, Berkshire SL1 5PR UK; 11 ARC Centre of Excellence in Plant Cell Walls and School of Agriculture, Food and Wine, The University of Adelaide, Waite Campus, Urrbrae, SA 5064 Australia; 12 Present Address: Vegetable Crop Research Unit, Keygene N.V, Agro Business Park, 90 6708 PW Wageningen, The Netherlands

**Keywords:** *Nicotiana glauca*, Metabolite profiling, Biorefinary, Bioproducts

## Abstract

The evaluation of plant-based feedstocks is an important aspect of biorefining. *Nicotiana glauca* is a solanaceous, non-food crop that produces large amounts of biomass and is well adapted to grow in suboptimal conditions. In the present article, compatible sequential solvent extractions were applied to *N. glauca* leaves to enable the generation of enriched extracts containing higher metabolite content comparing to direct leaf extracts. Typically, between 60 to 100 metabolite components were identified within the fractions. The occurrence of plant fatty acids, fatty acid alcohols, alkanes, sterols and terpenoids was detected by gas liquid chromatography–mass spectrometry (GC–MS) and metabolite identification was confirmed by comparison of physico-chemical properties displayed by available authentic standards. Collectively, co-products such waxes, oils, fermentable sugars, and terpenoids were all identified and quantified. The enriched fractions of *N. glauca* revealed a high level of readily extractable hydrocarbons, oils and high value co-products. In addition, the saccharification yield and cell wall composition analyses in the stems revealed the potential of the residue material as a promising lignocellulosic substrate for the production of fermentable sugars. In conclusion a multifractional cascade for valuable compounds/commodities has been development, that uses *N. glauca* biomass. These data have enabled the evaluation of *N. glauca* material as a potential feedstock for biorefining.

**Graphical Abstract:**

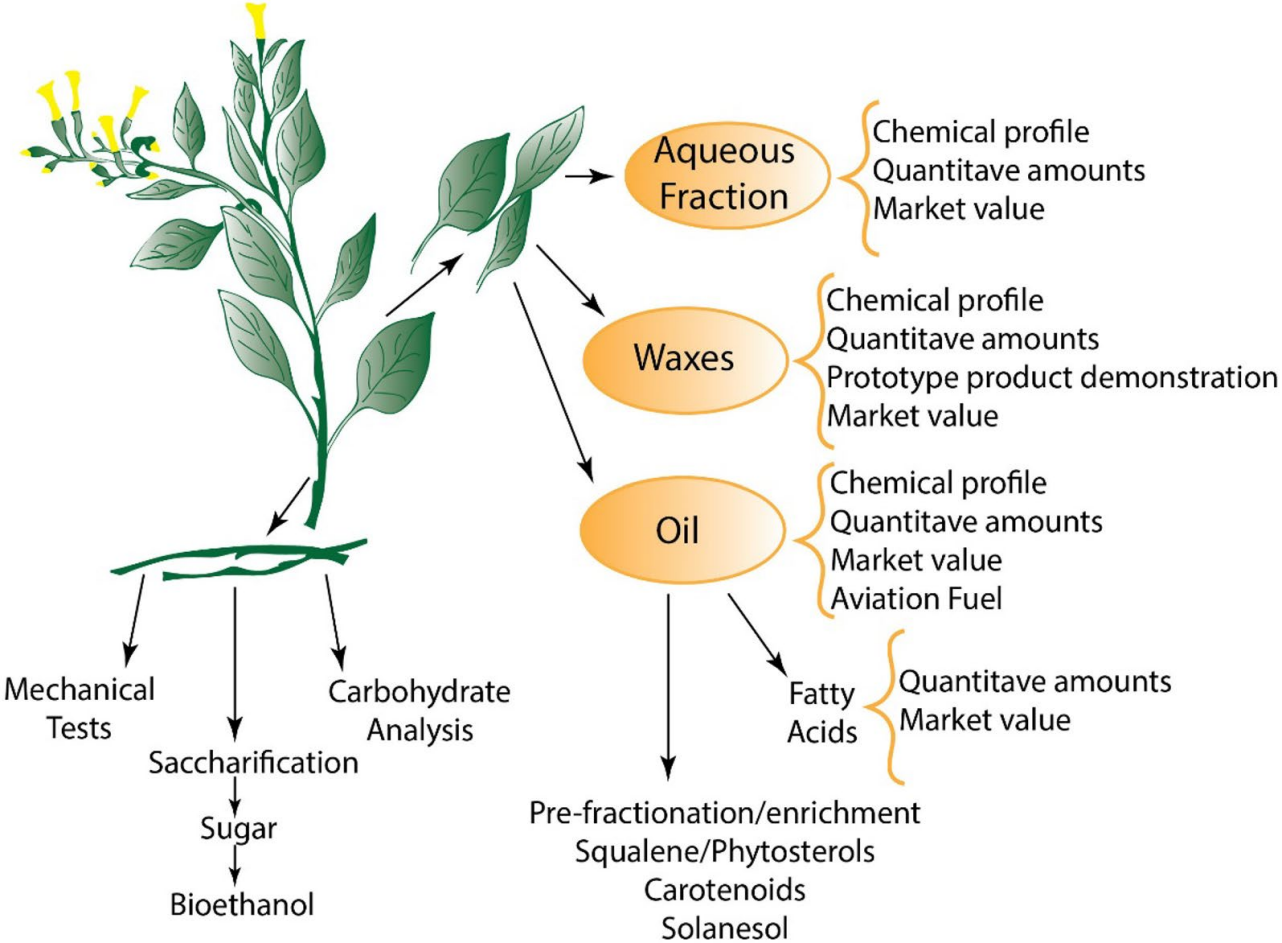

**Supplementary Information:**

The online version contains supplementary material available at 10.1186/s40643-023-00726-4.

## Introduction

At the beginning of the twentieth century, industrial economies were reliant on naturally occurring resources. The abundance of fossil fuels and associated technological advances in chemical refining led to socio-economic dependence on petrochemical refining for virtually all household goods, pharmaceuticals and fuels. However, increased energy insecurity, rising oil prices and the detrimental environmental impacts (pollution and climate change) of the industry, has led to an urgent need for renewable alternatives. The accelerating demands coincide with the need to avoid competition with food crops on arable land (IEA 2013).

*Nicotiana glauca* (*N. glauca*) or the “tobacco tree” is a fast-growing shrub that belongs to the Solanaceae family. It is native to central northwest Argentina and Bolivia, but it is widely distributed in temperate regions of Africa, Asia, Oceania and Europe (Curr and Fernández 1990; Florentine and Westbrooke 2005). Several traits reveal the potential of *N. glauca* as a potential feedstock for biorefining. The tobacco tree is a nicotine-free tobacco species which needs very low resource input, and it shows high levels of tolerance to both abiotic and biotic stresses. Moreover, it has a high sprouting capacity with up to 100% of the plant capable of sprouting resulting in the production of large quantities of above ground biomass. It is also easily grown in large scale cultivation on marginal lands/soils where there is no competition with the cultivation of food crops (Curt and Fernández 1990). The presence of soluble sugars has also previously attracted interest as a bioethanol source (Curt and Fernández 1990). Furthermore, *N. glauca* is amenable to transformation and thus development of modern synthetic biology resources and tools are easily implemented in this species (Mortimer et al. 2017). Although regarded as nicotine free *N. glauca* does contain anabasine which has a similar structure to nicotine but it is not as toxic or addictive. Potentially anabasine could be used as a precursor for new insecticidal structures.

In the present study, a range of biochemical and biophysical approaches were used to characterize leaf and stems materials from *N. glauca*. On a lab scale, a multifractional extraction cascade was carried out and the resulting extracts were assessed by GC–MS (gas chromatography–mass spectrometry)-based metabolite profiling. Further characterization of targeted compounds was carried out by LC–MS (liquid chromatography–mass spectrometry). Using this approach, a range of marketable, valuable chemicals were identified in *N. glauca* tissues. Compatible extraction cascades were devised resulting in metabolite-enriched extracts with industrial potential. In addition, biochemical analyses and micro-mechanical tests performed on the lignocellulosic biomass showed the material to be an effective substrate to produce fermentable sugars.

## Materials and methods

### Plant material

Wild-type plants of the tobacco tree *Nicotiana glauca* L. Graham variety were grown in field trials in the United Arab Emirates (UAE) in the summer 2013 and winter 2013, respectively. Fully expanded leaves from 8-week-old (mature) plants were harvested and air dried and were used for subsequent multi-fractional extractions. For every 100 plants cultivated kg quantities of biomass were generated.

Greenhouse grown plants were used for saccharification and cell wall analyses. Five biological replicates of wild-type plants of *Nicotiana glauca* L. Graham variety were grown from cuttings in a glasshouse under supplementary lightning. Stem samples were harvested after flowering and seed formation.

### Extraction methods for the generation of the aqueous, oil and wax fractions

Mature leaves of field trial-grown plants were air dried for 2 weeks in an air-conditioned room (approx. 21 °C) and subjected to three large scale extraction trials using ethanol and *n-*hexane as extraction solvents to optimize the extraction of the polar and non-polar fraction respectively.

#### Rapid extraction with *n*-hexane: non-polar extract 1

Approximately 300 g of dry leaves were placed into a heated double jacket flask with *n-*hexane (60% purity) and extracted for 10 min at 50 °C. Following the extraction, the solution containing the solvent and leaves was filtered with filter paper (Whatman qualitative filter paper grade 1), through a Bucher funnel, for 10 min. The extraction was repeated with *n*-hexane. The final filtered extract was then dried using a vacuum rotatory evaporator at 70 °C (Büchi Rotavapor 2-210), to generate the fraction referred to as non-polar extract 1.

#### Soxhlet extraction with n-hexane: non-polar extract 2

Approximately 251 g of dry leaves were recovered after the rapid *n-*hexane extraction. Surplus solvent in leaves was removed by air-drying overnight in an extractor hood. After drying, the leaves were milled to powder and placed in a Soxhlet equipment with 2.4 L. of *n-*hexane for 4 cycles of 255 min. Filtration was carried out in a fluted filter and the excess of solvent was removed by leaving the residue overnight in an extractor hood. The recovered extract was then dried using a vacuum rotatory evaporator at 70 °C to produce the fraction referred to as non-extract 2.

#### Soxhlet extraction with ethanol: aqueous fraction

Approximately 235.9 g of dry leaves were placed in a Soxhlet equipment with 2.4 L. of ethanol 75% for 3 cycles of 245 min. Filtration was carried out in fluted filter and surplus solvent was removed by drying overnight in an extractor hood. The recovered extract was then dried using a vacuum rotatory evaporator at 70 °C to generate the fraction referred to as aqueous fraction.

#### Soxhlet extraction with ethanol: wax fraction

Mature leaves from *N. glauca* were air dried and extracted in a Soxhlet equipment with ethanol (99%) for four cycles of 6 h. After extraction, the extract-ethanol solution was cooled down at 7 °C for 36 h. Substances with high melting point crystallized during cooling. The crystals were then separated by filtration and the wax extract was dried in an extractor hood to generate the fraction referred to as wax fraction.

### Extraction and analysis of metabolites

#### Extraction and GC–MS analysis of non-polar metabolites

Ten mg of non-polar extract 1 and 2 were saponified in 500 μl of methanol, 50 μl of KOH (60%, w/v) and incubated for 1 h at 50 °C. After cooling, the samples were extracted in 450 μl of H_2_O and 1 ml of chloroform containing the internal standard deuterated myristic acid) (D-27) (1 mg/ml). After centrifugation, the chloroform phase was transferred to an Eppendorf tube and 1 ml of chloroform was added for re-extraction. Subsequently, samples were derivatized to their methoxylated and silylated forms using methoxyamine hydrochloride (20 mg/ml in anhydrous pyridine) and *N*-methyl-*N*-trimethylsilyltrifluoroacetamide (MSTFA) (70 μl). Derivatised samples were injected in splitless mode into a 7890A GC online with 5975C mass spectrometer (Agilent Technologies). The chromatographic separation was performed using a DB-5MS 30 m × 250 μm column (J&W Scientific, Folsom) equipped with a 10 m guard column and using a temperature gradient from 70 °C to 320 °C at 5 °C/ min. Helium was employed as the carrier gas and the rate was set at 0.5 ml/min. The inlet was heated at 280 °C and the mass spectrometer transfer line at 250 °C. Mass spectra were recorded at 1.6 scans per second with a *m/z* 50–600 scanning range (Perez-Fons et al. 2014).

AMDIS software (version 2.7) was used for peak deconvolution and library search. Identification of compounds was done by comparison of mass spectra and retention indices to NIST (version 2.0) mass spectral database and in-house libraries built with authentic standards Compounds of interest such as hentriacontane (0.01–0.11 µg), squalene (0.01–0.06 µg) and linoleic acid (0.06–0.11 µg) were identified with authentic standards and quantified by comparison with dose–response curves (Mortimer et al. 2012).

#### Extraction and GC–MS analysis of polar metabolites

The extraction of the aqueous fraction of the material was essentially performed as described by (Lisec et al. 2006). Metabolite concentration in leaves and aqueous fraction was evaluated by relative concentration normalized by dry weight with extraction yield (w/w) of aqueous fraction in a multi-fractional extraction. Chromatograms and mass spectra were evaluated using TagFinder 4.0 (Luedemann et al. 2008) and Xcalibur 2.1 software (Thermo Fisher Scientific, Waltham, USA). Metabolites were identified in comparison to database entries of authentic standards (Kopka et al. 2005; Schauer et al. 2005).

#### Quantification of solanesol

The saponified non-polar phase obtained from the oil fractions was used for solanesol quantification. 100 µl of the chloroform phase obtained after the aforementioned non polar extraction were dried under vacuum and re-suspended in 50 μl of chloroform. Identification of solanesol was carried out using the high-resolution Q-TOF mass spectrometer UHR–MAXIS (Bruker Daltonics) on line with UHPLC UltiMate 3000 and equipped with a PDA detector (Dionex Softron). Chromatographic separations were performed as in (Fraser et al. 2000) and spectrometric parameters and detection as in (Jones et al. 2013). Quantification was carried out with dose–response curves (0.025–0.25 µg) using an authentic standard from Sigma-Aldrich (Fraser et al. 2000).

### Saccharification analysis

Five biological replicates of *Nicotiana glauca* L.Graham were grown from cuttings in a heated greenhouse. At the end of the life cycle (i.e., after flowering and seed formation) the stems of the plants were cut for processing. The stems were freeze-dried and the outer ‘bark’ layer was removed. The debarked stem was cut in small pieces and grinded using a Retsch mill MM300 until a particle size < 0.5 was obtained. The *N. glauca* samples were compared with stem samples of fully grown, senescent maize (*Zea mays* inbred line B104) and of 3-month-old greenhouse-grown poplar tree (*Populus tremula* × *Populus alba* cv. 717-1B4). 10 mg of biomass was saccharified with acid, alkali, ammonia and hot water pre-treatment as described in (Santoro et al. 2010; Van Acker et al. 2013). The ammonia pre-treatment was performed using 1 M NH4OH, incubating for 3 h at 90 °C.

### Cell wall polysaccharide analysis

Cell wall samples were prepared by grinding stems of *N. glauca* greenhouse-grown plants under liquid nitrogen to a fine powder. Identification and quantification of monosaccharides were performed using High Performance Anion-Exchange Chromatography with Pulse Amperometric Detection (HPAEC–PAD) and cellulose was extracted from ground plant material as described in (Lai-Kee-Him et al. 2002).

### Micromechanical tensile test

Micromechanical tests were performed on strips of the stems of three biological replicates of the *N. glauca* plants grown in the greenhouse. For sample preparation, the outermost region of the stem was removed and 100-*μ*m thick longitudinal–tangential (LT-) slices were cut using a rotatory microtome. After microtome cutting, strips with a width of 1.5 mm were prepared for testing using a scalpel. The strips were tested in tension in wet condition using a microtensile testing device equipped with a load cell with 50 N maximum load capacity (Burgert et al. 2003).

Real time strain detection was provided by a CCD video extensometry camera connected to a stereomicroscope. Initial test length was set to 14 mm. Samples were extended with a speed of 10 µm/s until rupture. The bulk density of the micromechanically tested samples was calculated based on green volume and oven dry mass (Rowell 2012).

## Results

### Metabolite profiling of enriched fractions

A two-step extraction process from leaves using *n*-hexane to generate non-polar extracts was evaluated. The first fraction (referred to as non-polar extract 1) was the product of a rapid *n*-hexane extraction, while a more exhaustive (i.e., Soxhlet) extraction was performed to produce a second oil-related extract (referred to as non-polar extract 2). In addition, an ethanol-based Soxhlet extraction was also performed to produce a refined wax (referred to as wax extract) and an aqueous extract (Additional file 1: Fig S1).

GC–MS-based metabolite profiling of non-polar extracts led to the identification of 45 compounds including plant fatty acids, fatty acid alcohols, alkanes, sterols and terpenoids (Table 1). Metabolite levels in those extracts obtained after multi-fractional processes were, in general, higher than those obtained from direct leaf extracts (Table 1 and Additional file 1: Table S1).

**Table 1:** Metabolites identified in the non-polar and polar fractions after multi-fractional extractions

	Compound class	Metabolites
Non polar fraction	Alkane	C_27_H_56_, C_29_H_60_, C_30_H_62_, C_31_H_64_, C_33_H_68_, C_25_H_52_, C_32_H_66_
	Free fatty acid	C12:0, C14:0, C15:0, C16:0, C17:0, C18:2 cis9,12 C18:3, C18:0, C20:0, C22:0, C24:0, C26:0
	Fatty acid methyl esther	C16:0, C18:3, C18:2 trans 9,12, C18:0, C20:0, C22:0, C24:0,
	Fatty acid alcohol	Tetracosanol, 1-hexadecanol, 1-octadecanol
	Glycerolipid	Glycero-2-C16:0, glycero-1-C18:0, glycero-1-C16:0
	Sterol	campesterol, stigmasterol, β-sitosterol
	Triterpenoid	Squalene
	Tocopherol	α-Tocopherol
Polar fraction	Amino acid	4-Hydroxyproline, arginine, aspartate, β-alanine, GABA, glutamate, glutamine, glycine, histidine, isoleucine, leucine, methionine, ornithine, phenylalanine, pyroglutamate, threonine, tyrosine, valine, slanine, asparagine, lysine, proline, serine, tryptophan
	Organic acid	Dehydroascorbate, fumarate, malate, quinate, succinate, threonate, 2-oxoglutarate, citrate,
	Sugar	Galactinol, glucose, raffinose, ribitol, sucrose, trehalose, fructose,
	Sugar acid	Glycerate
	Sugar alcohol	Erytritol, *myo-*inositol
	Other	Putrescine, tyramine, urea, benzoate, guanidine

List of metabolites that were detected and identified based upon similarity of mass spectra. For more exhaustive list and abundance of each of the identified metabolites referred to Additional file 1: Table S1 and S2

**Fig. 1:**
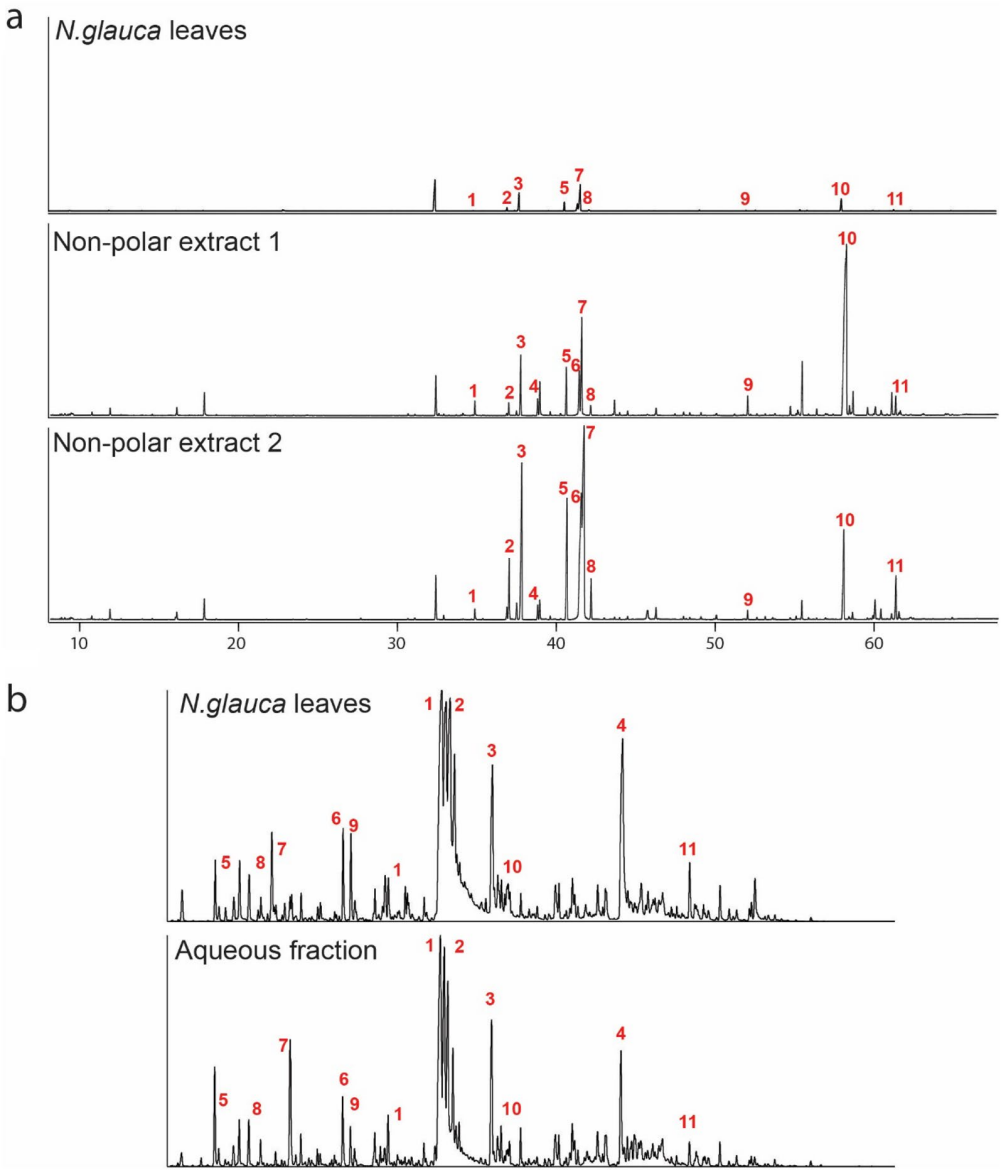
Gas chromatography profile of **a** non-polar metabolites of non-polar extracts1 and 2 and **b** polar metabolites derived from mature leaves of *N. glauca* after large scale extraction. ME: Methyl Esther. For non-polar metabolites numbers are: 1. C16:0 ME, 2. ALA 3TMS, 3. C16:0 ME, 4. C18:2 ME, 5. phytol, 6. C18:2, 7. ALA 3TMS, 8. C18:0, 9. tetracosanol-1-ol. 10. hentriacontane, 11. β-sitosterol. For Polar metabolites, numbers are: 1. fructose, 2. glucose, 3. *myo*-inositol, 4. sucrose, 5. glycerol, 6. malate, 7. succinate, 8. glycerate, 9. pyroglutamate, 10. galactose, 11. unknown sugar

Among the lipophilic compounds detected in multi-fractional non-polar extracts (i.e., non-polar extract 1 and non-polar extract 2), the abundance of the hydrocarbon hentriacontane (C31H64) predominated, representing 91.6% and 98.6% of the total amount of hydrocarbons identified in the non-polar extracts 1 and 2, respectively. In addition, the majority of hydrocarbons were recovered in both non-polar extracts, except for nonacosane, triacontane and hentriacontane which were detected at lower levels in non-polar extract 2. Other metabolites such as hexadecanoic acid, phytol, linoleic acid and linolenic acid, some isoprenoids and sterols such as squalene, stigmasterol, campesterol and β-sitosterol were also identified (Fig. 1a, Additional file 1: Table S1). Although most of the abundant compounds showed higher relative abundances in non-polar extract 2, other metabolites such as tetracosan-1-ol showed improved recovery rates in non-polar extract 1. Interestingly, most of the methyl esterified (ME) fatty acids had a higher recovery in non-polar extract 1 (Additional file 1: Table S1). These differences in recovery are of importance when applying the multi-fractional procedures for industrial applications to optimize the extraction of particular compounds.

Metabolite profiling was also applied to the wax fraction. The profile of this fraction consisted predominantly of long-chain hydrocarbons, such as nonacosane, triacontane, dotriacontane and tritriacontane. Interestingly, hentriancontane was by far the most abundant compound, suggesting that the implementation of this bioprocessing allows preferential enrichment of hentriacontane (Table 2, Additional file 1: Fig. S2).

**Table 2:** Quantification of selected metabolites in non-polar extracts 1,2 and aqueous fraction of mature leaves from *N. glauca* after multi-fractional process and direct leaf extracts

	Non polar fractions				Polar/Aqueous fraction				
	linoleic acid (mg/g)	Hentriacontane (mg/g)	Squalene (mg/g)	Solanesol (mg/g)	Glycerol (mg/g)	Fructose (mg/g)	Glucose (mg/g)	*myo*-inosito (mg/g)	Sucrose (mg/g)
*N. glauca* leaves	5.34	3.99	1.02	0.07	3.86	6.83	8.89	2.58	3.22
Non polar extract 1	31.43	236.88	6.5	1.41					
Non-polar extract 2	54.39	32.4	7.31	2.33					
Wax extract		202							
Aqueous fraction					1.97	9.67	2.04	1.90	1.10
Recovery yield non-polar extract 1 (%)	588.6	5936.8	637.3	1989.7					
Recovery yield non-polar extract 2 (%)	1018.5	812.0	716.7	3286.0					
Recovery yield wax (%)		5062.7							
Recovery yield aqueous fraction (%)					51	142	23	74	34

Concentrations are reported in mg/g of dry weight (DW). Recovery yield is expressed as percentage relative to the amount quantified in direct leaf extracts (*N. glauca* leaves)

### Metabolite composition of the aqueous fraction

The fraction produced after an ethanolic extraction in the multi-fractional process was aqueous in nature (Additional file 1: Fig. S1). To assess the presence of useful/valuable compounds in this fraction a non-targeted GC–MS-based polar metabolite profiling was performed. A total of 63 components were detected including sugars, sugar alcohols and amino acids (Table 1, Fig. 1b). Amino acids and sugars such as leucine, myo-inositol, galactinol, fructose, glucose, γ-amino butyric acid (GABA), pyroglutamate, sucrose and proline were detected in abundant amounts in direct leaf extracts (Additional file 1: Table S2). Furthermore, the profile of the enriched aqueous fraction compounds revealed that glycerol, valine, palmitate, leucine, malate, sucrose, galactinol, myo-inositol, fructose, glucose, proline, quinate, GABA, pyroglutamate were also present in significant levels. The metabolite contents were qualitatively similar compared to laboratory scale phase partitioning of leaf material. However, when normalised by extraction yield (w/w) following the multi-fractional approaches, dehydroascorbate, quinate, arginine, salicylic acid, putrescine, benzoate, citrate, sitosterol were present in higher yields (> tenfold higher amounts).

### Quantification of high value metabolites present in leaves of *N. glauca*

Of those compounds identified in the different fractions from the extracts prepared from *N. glauca* leaf materials fatty acids (i.e., C18:2) and long chain hydrocarbons (i.e. hentriacontane) were highly abundant. Squalene and solanesol, although not present in large quantities, are established commercially valuable products. To quantify the exact amounts of these compounds in the *N. glauca* fractions, calibration curves were prepared from authentic standards.

Linoleic acid and solanesol concentrations were higher in non-polar extract 2, while hentriacontane was higher and present in significant amounts in non-polar extract 1 and in the wax extract. The concentration of squalene was ~ sixfold higher in non-polar extracts comparing to the concentration in direct leaf extracts (Table 2). In general, the estimated concentrations in non-polar extracts were higher when compared to the leaf extract (Table 2), indicating the potential of compound enrichment obtained after bioprocessing.

The sugars contained in the aqueous extract was estimated using absolute quantification methods (Roessner-Tunali et al. 2003). The values reported in Table 2 show that glycerol, myo-inositol and sugars are present in high amounts with good recoveries, suggesting that *N. glauca* extracts can be used as fermentable natural sources for the generation of bioethanol, oil and other high value compounds.

### The potential production of other biorefinable products of *N. glauca*

Agricultural residues such as the stems of the plant also have potential use for the conversion of biomass into valuable chemicals. To evaluate the potential of *N. glauca* as lignocellulosic biomass, greenhouse-grown plants were used to perform saccharification and cell wall composition analyses. In addition, the stem density and mechanical properties of the xylem of *N. glauca* were tested.

For saccharification analysis the *N. glauca* samples were compared with stem samples of fully grown, senescent maize and 3-month-old poplars. Saccharification analysis showed that glucose release and cellulose conversion in *N. glauca* stems is higher when a hot water pre-treatment is applied, showing 7% of the cell wall converted into glucose, comparing to 4% and 6% in maize and poplar with the former being statistically significant (Additional file 1: Table S3, Fig S3). Pre-treatments are used to loosen up the plant cell wall, hence cell wall polysaccharides, especially cellulose become more accessible to the hydrolysing enzymes used for saccharification. The three biomass types were saccharified upon either no, an acid, two alkaline or a hot water pretreatment (Additional file 1: Table S3, Fig S3). The data show that for all three species, the alkaline pretreatments applied are most effective in preparing the biomass for saccharification. Additional file 1: Table S3 shows that poplar biomass performs better than that of *N. glauca* and maize without or with acid and ammonia pretreatments, and maize outperforms with the alkaline pretreatment. Upon hot water pretreatment, the saccharification efficiency of *N. glauca* biomass is as high as that of poplar biomass and better than that of maize biomass. The use of a hot water pre-treatment excludes the use of expensive and hazardous chemicals and highlights the environmental credentials of the process.

The compositional analysis of cell wall carbohydrates of three separate parts of the plant (bark, stem and inner pith) (Additional file 1: Table S4) revealed that cellulose is the dominant component, with cellulose levels comparable to those of other similar hardwood crops (Sannigrahi et al. 2010).

On the other hand, the mechanical properties of the xylem of *N. glauca* are comparable to that of oil palms which in principle makes it suitable for such composites (Additional file 1: Table S5). Further research is required to show that processing and incorporation of these fibres is feasible.

## Discussion

Plant biomass has for a long-term been investigated as a renewable source of energy and chemicals. Increasing efforts are being made to not only find sources of renewable feedstocks for industry, but also to improve existing crops through engineering (Fesenko and Edwards 2014). However, the rising demands for food production and the need to ensure food security will require plants to be cultivated in marginal unfertile lands, where abiotic environmental factors are suboptimal (Schippers et al. 2015).

In this study, an underutilised non-food crop has been explored as a potential plant feedstock for the biorefining industry. The proposed characterization for *N. glauca* involves an extraction cascade in which the different fractions are chemically enriched to different degrees and contain a range of useful and valuable compounds. Our results show that a simple refining process at large scale can be used to enrich chemical entities through complementary solvent extractions.

*Nicotiana glauca* represents an underutilised crop and potential feedstock production system for renewables. To further evaluate its potential, we have used a non-targeted metabolomics approach to evaluate the chemical profile of this plant. The lipophilic profile of *N. glauca* shows a typical composition of plant oils with major constituents being linoleic acid (C18:2) and palmitic acid (C16:0) that can be utilised for biofuel production (Durrett et al. 2008). The linoleic acid composition (~ 16% of the oil fraction) is comparable to that of plant species such as *Brassica rapa* seed content (~ 20%) (Borissova et al. 2010; Ramos et al. 2009), and it is increased by twofold following bioprocessing. The next challenge will be the evaluation of the large-scale adoption of *N. glauca* biofuel in sufficient amounts to supply industry demands.

In this study, chemical co-products that are currently in demand in the pharmaceutical industry were identified in *N. glauca* (i.e., solanesol and squalene). Solanesol is a naturally occurring triterpene alcohol with recognised antibiotic, antioxidant, and cardiac stimulating properties (Sarala et al. 2013; Srinivasa et al. 2014). It is also a source of isoprene units for the synthesis of high-value bio-products such as coenzyme Q10, vitamin K analogues, and the anticancer drug potentiating agent *N*-solanesyl*-N*, N1-bis (3,4-dimethoxy benzyl) ethylenediamine (Hamamura et al. 2002; ICAR 2006). The isoprenoids, including solanesol, have an estimated global market value of US$ 1 billion per annum (Taylor and Fraser 2011). The purification of solanesol has received special focus using tobacco leaf as starting material (Taylor and Fraset 2011) with the average price of solanesol (98% pure) ranging between US$550–US$1100 per kilogram (ICAR 2006). Reported values of solanesol content in tobacco leaf samples range broadly from 0.04 to 1.7% dry weight (Kotipalli 2008; Zhou and Liu 2006). In this study, after multi-fractional processing solanesol content in *N. glauca* leaves ranges from 0.14% to 0.23% dry weight (Table 2), which is in the lower range of the reported values for industrial tobacco leaf material (Kotipalli 2008; Zhou and Liu 2006). It should be noted, however, that the extraction procedures that were used are not dedicated to its enrichment, and therefore, a compromise is expected. The rising demand of this compound and the economic possibilities to add value to the crop open a new opportunity to investigate purification methods from other solanaceous plants (such as nicotine-free *N. glauca*), avoiding sources associated with the smoking industry.

The high quantities of hentriacontane present in *N. glauca* leaves and fractions highlight this plant as a readily valuable biosource of plant lipids in the form of wax. In addition, compounds such as squalene, which is an intermediate of the biosynthesis of phytosterols, are of high interest as the primary source of this compound is shark liver and the search for natural sources is a pressing demand. Our results show that the concentrations of squalene in mature leaves of *N. glauca* fall below the values reported for *Amaranthus* sp. (60–80 mg/g) (Popa et al. 2015), which is described as containing the highest concentration of squalene. However, they are comparable to the concentrations reported for other traditional natural sources such as olive oil (Popa et al. 2015).

The fermentable sugars are potentially useful compounds in the aqueous fraction. However, it is important to note that GABA, glutamate and sitosterol should additionally be considered. GABA is sold as a dietary supplement and its derivatives are used as tranquilizing agents (Lambertucci et al. 2012; Lapin 2001), glutamate is used as a taste enhancer and also as part of the oral supplement to reverse muscle wasting, while sitosterol is being studied for its potential to reduce benign prostatic hyperplasia and blood cholesterol levels (Assmann et al. 2006; Kim et al. 2012). Furthermore, salicylate is the active ingredient in aspirin-like pain relief tablets. However, the cheapness of its chemical synthesis means that it is not of high value, unless utilized as a co-product as illustrated in the present study.

Although not analyzed in this study, it is important to note that *N. glauca* is also characterized by the presence of high levels of pyridine alkaloids such as anabasine in leaf and root tissues. These alkaloids have been found to be an effective deterrent against herbivores (Saitoh et al. 1985; Sinclair et al. 2004). In addition, previous studies have reported the existence of Vitamin D_3_ compounds in *N. glauca* leaf tissue (Skliar et al. 2000).

Growing efforts are also focused on investigating the potential of lignocellulosic feedstock for and the production of fermentable sugars e.g. biofuel production. Evaluation of the sugar content with respect to its potential use for bioethanol conversion as well as cellulose to sugars via saccharification conversion has been carried out and benchmarked the outputs to maize and poplar which are commonly known and accepted bioenergy crops. Although the saccharification efficiencies under no, acid and alkaline pretreatments were lower for *N. glauca* than those for poplar and maize biomass, the *N. glauca* biomass performed equally well as poplar and better than maize upon hot water pre-treatment. Although the saccharification efficiencies upon hot water pretreatment are lower comparing to acid and alkaline pretreatments (Additional file 1: Table S3), this pre-treatment excludes the use of expensive and hazardous chemicals and highlights the environmental credentials of the process. *Phaffia rhodozyma* is an example of a non-conventional yeast that can utilise sugars from lignocellulose breakdown, producing the high value carotenoid astaxanthin and ethanol due to the metabolic use of the “Crabtree effect” (Reynders et al. 1997).

The compositional cell wall polysaccharide analyses show that the cellulose content in *N. glauca* is comparable to other hardwood species and that the crystallinity of cellulose in *N. glauca* stems is higher than published data on poplar (Sun et al. 2014a; Sun et al. 2014b; Liu et al. 2012).

On the other hand, the mechanical properties of the xylem of *N. glauca* are comparable to that of oil palms which in principle makes it suitable for such composites. Further research would have to show that processing and incorporation of these fibres is feasible.

## Conclusions

Imbedded in the success of using plant biomass as renewable feedstocks is the application of modern biotechnology to improve the production of the desire chemicals (Fesenko and Edwards 2014; Stewart 2007; Wolt 2009). However, concerns of employing genetic modification for industrial purposes creates extensive biosafety and regulatory issues (Stewart 2007). A key advantage of *N. glauca* is that it is not related to food crops and is readily amenable for genetic transformation. Improving the output traits in a non-food crop that easily grows in marginal land is an attractive one.

The approach described in this study shows how metabolite profiling can be incorporated into the discovery phase of biorefining approaches to identify and optimise enrichment of multiple co-products that collectively add value to a non-food crop that will propagate on non-fertile land. The data generated provide the foundations for future TechnoEcomonic Analysis (TEA) and Life Cycle Analysis (LCA).

### Supplementary Information

**Additional file 1: Figure S1.** Schematic diagram of extraction procedures used to enrich metabolites for tobacco tree biomass. **Figure S2.** Relative metabolite content in the wax fraction of mature leaves *N. glauca.* **Figure S3.** Effect of a pre-treatment after 72 h of saccharification, including glucose release per dry weight, glucose release per cell wall residue (CWR) and cellulose conversion for *N. glauca,* maize and poplar. Bars represent averages of 5 biological replicates, error bars are standard deviations. * indicates significant changes compared to maize and † indicates significant changes compared to poplar within each pre-treatment (*T* test, *P* < 0.05). **Table S1.** Relative abundance of non-polar metabolites detected in non-polar extract 1 and 2 of mature leaves of *N. glauca* by GC–MS analysis. Metabolites were identified partly following the metabolomics reporting standards [1]. Relative abundance was evaluated by normalization. **Table S2.** Polar metabolites detected in aqueous fraction of mature leaves of *N. glauca* by GC–MS analysis. Metabolites were identified in comparison to database entries of authentic standards (Kopka et al 2005; Schauer et al 2005). Relative abundance was evaluated by normalization. **Table S3.** Glucose releases, expressed as percentage dry weight (DW) or percentage cell wall residue (CWR), after 72 h of saccharification. Based on the measured crystalline cellulose contents, the cellulose conversion could be calculated. Values are the means of 5 biological replicates ± SD. Values in bold correspond to the highest value in each treatment. *T* test comparisons between *N. glauca* and maize (*) and *N. glauca* and poplar (†) represent statistical significance at the 0.05 threshold. **Table S4.** Cell wall composition and cellulose crystallinity in *N. glauca* tissues. Composition values are presented as µg of component per mg of dry cell wall, where cell wall material does not include ash, proteins or other extractives. Values are the means for six biological replicate samples, each analysed in duplicate ± SD. Cellulose crystallinity index (CrI) values were calculated from XRD data shown in Fig. S4. Fuc: Fucose, Ara: Arabinose, Rha: Rhamnose, Gal: Galactose, Glc: Glucose, Xyl: Xylose. **Table S5**. Average values and standard deviation of mechanical Properties of xylem of mature stems of *N. glauca *extracted from micro-tensile tests of tissue slices (*n* = 33).

## Data Availability

We have provided data at a high level of disclosure however raw data and materials are available on request to the corresponding author.
