# Supplementary material for: Non-targeted discovery of high-value bio-products in Nicotiana glauca L: a potential renewable plant feedstock
Source: Bioresour Bioprocess. 2024 Jan 18;11(1):12. doi: 10.1186/s40643-023-00726-4 (PMC10991672; doi:10.1186/s40643-023-00726-4)
Supplement: Supplementary file 1 — Additional file 1: Figure S1. Schematic diagram of extraction procedures used to enrich metabolites for tobacco tree biomass. Figure S2. Relative metabolite content in the wax fraction of mature leaves N. glauca. Figure S3. Effect of a pre-treatment after 72 h of saccharification, including glucose release per dry weight, glucose release per cell wall residue (CWR) and cellulose conversion for N. glauca, maize and poplar. Bars represent averages of 5 biological replicates, error bars are standard deviations. * indicates significant changes compared to maize and † indicates significant changes compared to poplar within each pre-treatment (T test, P < 0.05). Table S1. Relative abundance of non-polar metabolites detected in non-polar extract 1 and 2 of mature leaves of N. glauca by GC–MS analysis. Metabolites were identified partly following the metabolomics reporting standards [1]. Relative abundance was evaluated by normalization. Table S2. Polar metabolites detected in aqueous fraction of mature leaves of N. glauca by GC–MS analysis. Metabolites were identified in comparison to database entries of authentic standards (Kopka et al 2005; Schauer et al 2005). Relative abundance was evaluated by normalization. Table S3. Glucose releases, expressed as percentage dry weight (DW) or percentage cell wall residue (CWR), after 72 h of saccharification. Based on the measured crystalline cellulose contents, the cellulose conversion could be calculated. Values are the means of 5 biological replicates ± SD. Values in bold correspond to the highest value in each treatment. T test comparisons between N. glauca and maize (*) and N. glauca and poplar (†) represent statistical significance at the 0.05 threshold. Table S4. Cell wall composition and cellulose crystallinity in N. glauca tissues. Composition values are presented as µg of component per mg of dry cell wall, where cell wall material does not include ash, proteins or other extractives. Values are the means for six biological [file 40643_2023_726_MOESM1_ESM.docx]

**Additional file**

**Figure S1.** Schematic diagram of extraction procedures used to enrich metabolites for tobacco tree biomass.

**Figure S2**. Relative metabolite content in the wax fraction of mature leaves *N. glauca.*


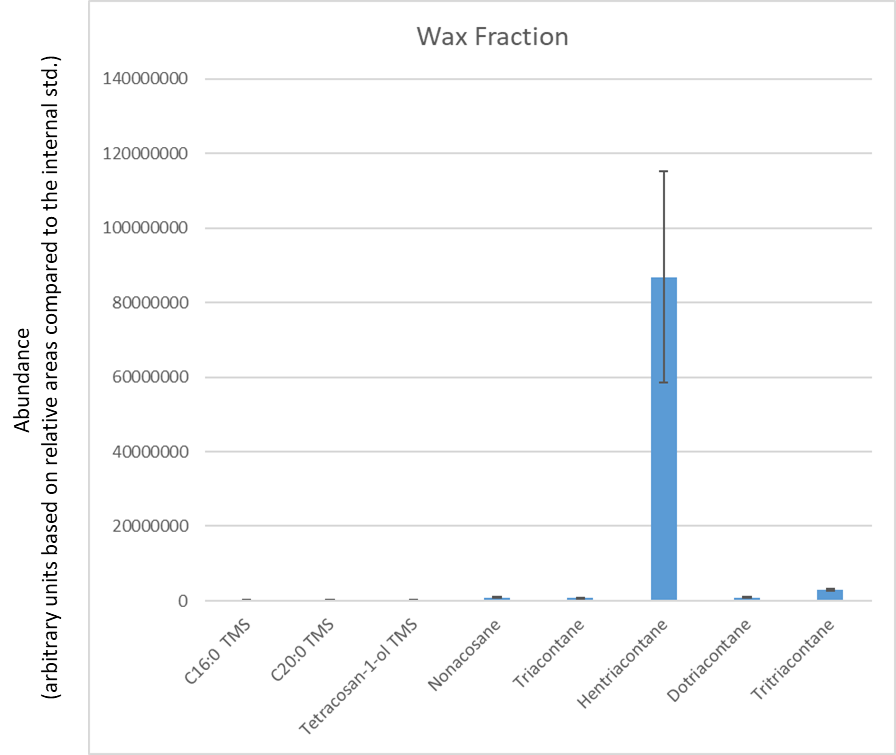

**Figure S3**. Effect of a pre-treatment after 72 hours of saccharification, including glucose release per dry weight, glucose release per cell wall residue (CWR) and cellulose conversion for *N. glauca,* maize and poplar. Bars represent averages of 5 biological replicates, error bars are standard deviations. * indicates significant changes compared to maize and ⴕ indicates significant changes compared to poplar within each pre-treatment (T-test, P<0.05).

**Table S1.** Relative abundance of non-polar metabolites detected in non-polar extract 1 and 2 of mature leaves of *N.glauca* by GC-MS analysis. Metabolites were identified partly following the metabolomics reporting standards [[1](#_ENREF_1)]. Relative abundance was evaluated by normalization.

| **Metabolite** | **leaves** | **non-polar extract 1** | **non-polar extract 2** |
| --- | --- | --- | --- |
| Phosphate | 7159.95 | 324135 | 925559.3 |
| Glyceric acid 3TMS | 7159.95 | 13954.4 | 30450.806 |
| Erythronic acid 4TMS | 7159.95 | 13954.4 | 34129.91 |
| C12:0 1TMS | 7159.95 | 13954.4 | 523602.28 |
| Glycerol-3-P | 7159.95 | 558732 | 374168.92 |
| C14:0 TMS | 280535 | 462857 | 1391653.9 |
| C16:0 ME | 859639 | 5176883 | 3516641.4 |
| C15:0 1TMS | 7159.95 | 104496 | 303320.35 |
| alpha linolenic acid ME | 7317772 | 5148769 | 22237431 |
| C16:0 1TMS | 4.2E+07 | 2.6E+07 | 87378425 |
| C18:2 trans9,12 ME | 7159.95 | 6493167 | 4539427.2 |
| C18:0 ME | 881599 | 1131570 | 1088717.6 |
| C17:0 1TMS | 7159.95 | 110335 | 353846.78 |
| Phytol (trans) 1TMS | 2E+07 | 2.1E+07 | 57481813 |
| C18:2 cis9,12 TMS | 2.3E+07 | 2.7E+07 | 66652885 |
| alpha linolenic acid 3TMS | 8.2E+07 | 5.1E+07 | 161485989 |
| C18:0 1TMS | 4726714 | 4846498 | 13831611 |
| C20:0 ME | 7159.95 | 1257989 | 454203.79 |
| C20:0 (arachidic acid) 1TMS | 907171 | 3979740 | 4256667.6 |
| C25H52, Pentacosane | 7159.95 | 574073 | 5550.9425 |
| C22:0 ME | 7159.95 | 551223 | 186240.22 |
| Glycero-2-C16:0 2TMS | 169586 | 50434.9 | 5550.9425 |
| Glycero-1-C16:0 2TMS | 3146763 | 1360350 | 784411.4 |
| C22:0 (behenic acid) 1TMS | 105964 | 1167665 | 1778296.1 |
| C27H56, Heptacosane | 7159.95 | 505488 | 5550.9425 |
| C24:0 ME | 7159.95 | 151163 | 115657.25 |
| Tetracosan-1-ol 3TMS | 1323441 | 8438743 | 2874956.5 |
| Glycero-1-C18:0 2TMS | 2698176 | 993193 | 600756.18 |
| Squalene | 59309.5 | 503599 | 911380.38 |
| C24:0 TMS | 7072.17 | 251179 | 632382.29 |
| C29H60, Nonacosane | 7159.95 | 3755754 | 414464.58 |
| Silane, (hexacosyloxy)trimethyl (1-Hexadecanol, O-TMS) | 2717168 | 2.4E+07 | 6744935 |
| UNKnp56.03min | 2571437 | 524031 | 635208.29 |
| C30H62, Triacontane | 7159.95 | 2706147 | 151089.66 |
| C26:0 1TMS | 7159.95 | 1173550 | 458445.02 |
| C31H64, Hentriacontane | 3.1E+07 | 2.2E+08 | 45175491 |
| Tocopherol-alfa 1TMS | 195496 | 310426 | 758274.79 |
| Silane trimethyl(octacosysyloxy)(1-Octadecanol, O-TMS) | 691610 | 9671362 | 2328534 |
| C32H66, Dotriacontane | 7159.95 | 3095792 | 5550.9425 |
| Campesterol 1TMS | 1327139 | 4579043 | 8042193.9 |
| Stigmasterol 1TMS | 508020 | 1989679 | 3628544.1 |
| Tocopherolhydroquinone alfa 3TMS | 7159.95 | 286753 | 35896.718 |
| C33H68, Tritriacontane | 7159.95 | 9183715 | 5550.9425 |
| beta-Sitosterol 1TMS | 2999068 | 8570884 | 17567777 |
| Tocopherol-alfa 1TMS | 7159.95 | 97102.6 | 35071.487 |

1. Sumner LW, Amberg A, Barrett D, Beale MH, Beger R, Daykin CA, Fan TWM, Fiehn O, Goodacre R, Griffin JL *et al*: **Proposed minimum reporting standards for chemical analysis Chemical Analysis Working Group (CAWG) Metabolomics Standards Initiative (MSI)**. *Metabolomics : Official journal of the Metabolomic Society* 2007, **3**(3):211-221.

**Table S2**. Polar metabolites detected in aqueous fraction of mature leaves of *N.glauca* by GC-MS analysis. Metabolites were identified in comparison to database entries of authentic standards (Kopka *et al* 2005, Schauer *et al* 2005). Relative abundance was evaluated by normalization

| **Metabolite** | **Leaves** | **Aqueous fraction** |
| --- | --- | --- |
| 1,6-hydro-glucose | 14933 | 51678 |
| 2oxglutarate | 0 | 32686 |
| 4-hydroxy-benzoate | 2366 | 21265 |
| 4-hydroxyproline | 23377 | 38275 |
| Adenine | 394264 | 703010 |
| Alanine | 44437 | 34862 |
| Arginine | 463 | 8274 |
| Asparagine | 117501 | 128477 |
| Aspartate | 34646 | 33255 |
| Benzoate | 6920 | 330450 |
| beta-alanine | 9147 | 22352 |
| Citrate | 1430 | 72468 |
| Dehydroascorbate | 3769 | 38891 |
| Erythritol | 163759 | 694977 |
| Fumarate | 28672 | 64294 |
| Fructose | 1815034 | 2895848 |
| GABA | 1897586 | 5023942 |
| Galactinol | 1361740 | 2758431 |
| Glucose | 1880980 | 3481478 |
| Glutamate | 56178 | 10911 |
| Glutamine | 29708 | 13322 |
| Glycerate | 45284 | 296374 |
| Glycerol | 572546 | 1298792 |
| Glycerol-3-phosphate | 7685 | 20297 |
| Glycine | 125477 | 207462 |
| Guanidine | 4164 | 9820 |
| Guanine | 47182 | 44873 |
| Guanosine | 134896 | 295201 |
| Histidine | 33262 | 2103 |
| Isoleucine | 463725 | 806289 |
| Leucine | 1190358 | 1743827 |
| Lysine | 157053 | 3092 |
| Malate | 413362 | 1812488 |
| Methionine | 5815 | 850 |
| Myo-inositol | 1198446 | 2852771 |
| Nicotinate | 52120 | 322513 |
| Octadecanoate | 26068 | 58373 |
| Ornithine | 33816 | 56943 |
| Palmitate | 331052 | 1509600 |
| Phenylalanine | 441781 | 664168 |
| Phosphorate | 135935 | 244940 |
| Proline | 5890745 | 4114481 |
| Putrescine | 888 | 23451 |
| Putrescine | 62458 | 33387 |
| Pyroglutamate | 2104539 | 16528617 |
| Quinate | 433254 | 4580639 |
| Raffinose | 510982 | 308113 |
| Ribitol | 203690 | 151371 |
| Ribulose-5-phosphate | 4133 | 26087 |
| Salicilate | 279 | 6198 |
| Serine | 301939 | 422004 |
| Sitosterol | 7 | 15815 |
| Succinate | 50948 | 334597 |
| Sucrose | 2612947 | 2561342 |
| Threonate | 66881 | 350569 |
| Threonine | 187163 | 240514 |
| Trehalose | 58471 | 251031 |
| Tryptophan | 325064 | 29974 |
| Tyramine | 129119 | 274429 |
| Tyrosine | 45552 | 89275 |
| Uracil | 22208 | 116201 |
| Urea | 170298 | 880866 |
| Valine | 753278 | 1380177 |

Kopka, J., et al. (2005). GMD@CSB.DB: the Golm Metabolome Database. Bioinformatics 21, 1635-1638 doi:10.1093/bioinformatics/bti236

Schauer, N., et al. (2005). GC–MS libraries for the rapid identification of metabolites in complex biological samples. FEBS Letters 579, 1332-1337 doi:<http://dx.doi.org/10.1016/j.febslet.2005.01.029>

**Table S3**: Glucose releases, expressed as percentage dry weight (DW) or percentage cell wall residue (CWR), after 72 hours of saccharification. Based on the measured crystalline cellulose contents, the cellulose conversion could be calculated. Values are the means of 5 biological replicates ± SD. Values in bold correspond to the highest value in each treatment. T-test comparisons between *N. glauca* and maize (*) and *N. glauca* and poplar (ⴕ) represent statistical significance at the 0.05 threshold.

| Plant material | Pretreatment | Glucose release  (% DW) | Glucose release (% CWR) | Cellulose conversion (% cellulose) |
| --- | --- | --- | --- | --- |
|  |  | after 72 hours | after 72 hours | after 72 hours |
| *N. glauca* | None | 3.42^ⴕ^ ± 1.22 | 5.02^ⴕ^ ± 1.98 | 14.44^ⴕ^ ± 5.55 |
| Maize |  | 3.39 ± 0.15 | 5.39 ± 0.28 | 16.49 ± 1.59 |
| Poplar |  | **6.09 ± 1.27** | **7.65 ± 1.61** | **24.26 ± 1.65** |
| *N. glauca* | Acid | 4.92*^ⴕ^ ± 0.86 | 7.17*^ⴕ^ ± 1.57 | 20.64*^ⴕ^ ± 4.17 |
| Maize |  | 6.04 ± 0.33 | 9.60 ± 0.41 | 29.39 ± 2.55 |
| Poplar |  | **8.21 ± 0.68** | **10.32 ± 0.83** | **29.84 ± 3.21** |
| *N. glauca* | Alkali | 9.59*^ⴕ^ ± 1.54 | 13.96*^ⴕ^ ± 2.81 | 40.2*^ⴕ^ ± 7.24 |
| Maize |  | **18.92 ± 0.44** | **30.12 ± 1.26** | **92.19 ± 8.40** |
| Poplar |  | 12.65 ± 1.05 | 15.91 ± 1.37 | 45.97 ± 5.08 |
| *N. glauca* | Ammonia | 6.88*^ⴕ^ ± 1.03 | 10.00^ⴕ^ ± 1.89 | 28.41^ⴕ^ ± 0.63 |
| Maize |  | 5.91 ± 0.98 | 9.38 ± 1.32 | 28.67 ± 4.18 |
| poplar |  | **10.68 ± 0.50** | **13.43 ± 0.62** | **38.82 ± 3.63** |
| *N. glauca* | Hot water | 4.82* ± 1.24 | **7.06* ± 2.12** | **20.31* ± 5.87** |
| Maize |  | 2.75 ± 0.17 | 4.38 ± 0.18 | 13.37 ± 0.65 |
| Poplar |  | **4.96 ± 0.59** | 6.23 ± 0.71 | 18.09 ± 3.21 |

**Table S4.** Cell wall composition and cellulose crystallinity in *N. glauca* tissues. Composition values are presented as µg of component per mg of dry cell wall, where cell wall material does not include ash, proteins or other extractives. Values are the means for six biological replicate samples, each analysed in duplicate ± SD. Cellulose crystallinity index (CrI) values were calculated from XRD data shown in Supplementary Fig 4. Fuc: Fucose, Ara: Arabinose, Rha: Rhamnose, Gal: Galactose, Glc: Glucose, Xyl: Xylose.

| Tissue | Fuc | Ara | Rha | Gal | Glc  (non-cellulose) | Xyl | Man | GalA | (Me) GlcA | Glc (cellulose) | Cellulose crystallinity  (CrI) |
| --- | --- | --- | --- | --- | --- | --- | --- | --- | --- | --- | --- |
| Stem | 0.4 ± 0.3 | 3.5 ± 1.1 | 2.4 ± 0.8 | 5.1 ± 1.2 | 9.9 ± 2.7 | 49.6 ± 9.5 | 2.5 ± 0.5 | 0.5 ± 0.2 | 0.4 ± 0.2 | 95.6 ± 18.8 | **74.0%** |
| Bark | 0.3 ± 0.2 | 19.1 ± 5.2 | 5.1 ± 0.8 | 15.5± 2.4 | 12.2 ± 7.7 | 10 ± 4.2 | 1.7 ± 0.9 | 3.0 ± 0.9 | 0,6 ± 0.1 | 63.3 ± 30.2 | **73.0%** |
| Pith | 0.2 ± 0.1 | 13.6 ± 2.6 | 6.7 ± 1.1 | 27.3 ± 4.4 | 12.7 ± 3.2 | 2.9 ± 0.8 | 0.9 ± 0.2 | 6.3 ± 1.9 | 1.1 ± 0.2 | 52.6 ± 19.7 | **72.5%** |

**Table S5**. Average values and standard deviation of mechanical Properties of xylem of mature stems of *N.glauca .*extracted from micro-tensile tests of tissue slices (n=33)

| E-Modulus (GPa) | 2.9±0.8 |
| --- | --- |
| Ultimate Stress (MPa) | 60.4±9.8 |
| Ultimate Strain (-) | 0.028±0.006 |
| Yield Strength (MPa)  (n=17) | 39.0±7.5 |
| Yield Strain (-)  (n=17) | 0.015±0.003 |
| Toughness (J.m^-3^.10^4^) | 1.0±0.3 |
| Raw Density (g/cm^3^) at 12% wmc | 0.38±0.03 |
